# Highly differentiated loci resolve phylogenetic relationships in the Bean Goose complex

**DOI:** 10.1186/s12862-023-02103-3

**Published:** 2023-01-19

**Authors:** Jente Ottenburghs, Johanna Honka, Marja E. Heikkinen, Jesper Madsen, Gerhard J. D. M. Müskens, Hans Ellegren

**Affiliations:** 1 Department of Evolutionary Biology, University of Uppsala, Uppsala, Sweden; 2 Ecology and Genetics Research Unit, University of Oulu, PO Box 3000, 90014 Oulu, Finland; 3 Department of Ecoscience, Aarhus University, C. F. Møllers Allé 8, 8000 Aarhus C, Denmark; 4 Team Animal Ecology, Wageningen Environmental Research, Wageningen University & Research, Droevendaalsesteeg 3-3A, 6708 PB Wageningen, The Netherlands

**Keywords:** Anseriformes, Introgression, Differentiation islands, Phylogenomics, Species tree

## Abstract

**Background:**

Reconstructing phylogenetic relationships with genomic data remains a challenging endeavor. Numerous phylogenomic studies have reported incongruent gene trees when analyzing different genomic regions, complicating the search for a ‘true’ species tree. Some authors have argued that genomic regions of increased divergence (i.e. differentiation islands) reflect the species tree, although other studies have shown that these regions might produce misleading topologies due to species-specific selective sweeps or ancient introgression events. In this study, we tested the extent to which highly differentiated loci can resolve phylogenetic relationships in the Bean Goose complex, a group of goose taxa that includes the Taiga Bean Goose (*Anser fabalis*), the Tundra Bean Goose (*Anser serrirostris*) and the Pink-footed Goose (*Anser brachyrhynchus*).

**Results:**

First, we show that a random selection of genomic loci—which mainly samples the undifferentiated regions of the genome—results in an unresolved species complex with a monophyletic *A. brachyrhynchus* embedded within a paraphyletic cluster of *A. fabalis* and *A. serrirostris*. Next, phylogenetic analyses of differentiation islands converged upon a topology of three monophyletic clades in which *A. brachyrhynchus* is sister to *A. fabalis*, and *A. serrirostris* is sister to the clade uniting these two species. Close inspection of the locus trees within the differentiated regions revealed that this topology was consistently supported over other phylogenetic arrangements. As it seems unlikely that selection or introgression events have impacted all differentiation islands in the same way, we are convinced that this topology reflects the ‘true’ species tree. Additional analyses, based on D-statistics, revealed extensive introgression between *A. fabalis* and *A. serrirostris*, which partly explains the failure to resolve the species complex with a random selection of genomic loci. Recent introgression between these taxa has probably erased the phylogenetic branching pattern across a large section of the genome, whereas differentiation islands were unaffected by the homogenizing gene flow and maintained the phylogenetic patterns that reflect the species tree.

**Conclusions:**

The evolution of the Bean Goose complex can be depicted as a simple bifurcating tree, but this would ignore the impact of introgressive hybridization. Hence, we advocate that the evolutionary relationships between these taxa are best represented as a phylogenetic network.

**Supplementary Information:**

The online version contains supplementary material available at 10.1186/s12862-023-02103-3.

## Background

Reconstructing the Tree of Life remains one of the major goals in evolutionary biology [1]. The advent of genomic data ushered in careful optimism to resolve some phylogenetically challenging questions, such as deep branching patterns and rapid adaptive radiations, ultimately converging upon a species tree (i.e. a phylogenetic tree that follows the branching pattern of consecutive speciation events, ref. [2]). However, analyses of multiple genes have revealed widespread discordance among gene trees in many different lineages [3]. In other words, different genes tell different evolutionary stories. This phylogenetic incongruence can be due to several biological processes, such as incomplete lineage sorting, hybridization or gene duplication [2, 4], and led to the development of methods to estimate a species tree from a collection of discordant gene trees [5–9].

Understanding the underlying processes responsible for gene tree incongruence can inform phylogenetic analyses and the choice of molecular markers. For example, a recent study reconstructed the phylogeny of the cat family (Felidae) while taking into account variation in recombination rate across the genome [10]. They showed that the phylogenetic signal for the species tree was concentrated within regions of low recombination, whereas regions of high recombination were heavily influenced by ancient gene flow. Possibly, high-recombining regions will more effectively remove alleles introduced by hybridization while loci contributing to reproductive isolation accumulate in low-recombining regions [11, 12]. Moreover, regions of low recombination tend to have lower effective population sizes, reducing the confounding effects of incomplete lineage sorting on reconstructing phylogenetic relationships [13]. Consequently, regions of low recombination might retain ancient branching events in the presence of interspecific gene flow [10, 14].

Because local recombination rates are not always available, other population genetic measures can be used to inform phylogenetic analyses. For example, given that regions of low recombination are generally more differentiated, regions of high genetic differentiation can potentially guide phylogenetic analyses. Genome scans have shown that differentiation varies across the genome and is often concentrated in particular “islands of differentiation” [15, 16]. The processes responsible for the emergence of these islands are still a matter of debate [17, 18]. Currently, two main theories attempt to explain the formation of differentiation islands. First, they might house loci involved in reproductive isolation whereas the rest of the genome remains undifferentiated by inter- or intraspecific gene flow [19–21]. Second, these differentiated islands might be the outcome of reduced genetic diversity due to linked selection as the reduction in genetic diversity in one species can contribute to increased genetic differentiation with other species that did not experience selection [22–26].

Regardless of the underlying process, genomic islands of differentiation are promising candidates to resolve complex phylogenetic relationships because of their increased divergence. Indeed, some authors have argued that islands of differentiation are more likely to reflect the species tree [18, 27]. In a speciation-with-gene-flow model, differentiation islands contain loci involved in reproductive isolation. Selection against introgression is thought to maintain the species tree whereas introgression masks the topology at other loci. In the context of linked selection, trees constructed from differentiation islands are expected to reflect the species tree because selection will reduce the effective population size, thereby accelerating the lineage sorting process. However, it has been shown that divergent genomic regions can also produce misleading tree topologies due to selection or introgression [28, 29]. For example, in a phylogenomic study on black-and-white flycatchers (genus *Ficedula*), Nater et al. (2015) found a large variety of tree topologies within differentiation islands, of which the most common topology deviated from the top-ranking topology obtained genome-wide. This incongruence can be explained by species-specific selective sweeps, resulting in patterns of genetic divergence that conflict with the species tree [30]. Similarly, Zhang et al. [29] reported tree topologies that deviated from the species tree in differentiation islands of *Phylloscopus* warblers, which they attributed to ancient introgression (also see [31]). These examples clearly indicate that the use of differentiation islands in phylogenomic analyses should be approached with caution.

In this study, we explore the extent to which highly differentiated genomic loci can resolve the phylogenetic relationships within the Bean Goose complex. This species complex is comprised of several taxa of which the taxonomic status is still a matter of debate. We will follow the classification of the International Ornithological Congress (IOC) Bird List, which recognizes three species: the Taiga Bean Goose (*Anser fabalis,* with three subspecies), the Tundra Bean Goose (*Anser serrirostris*, with two subspecies) and the Pink-footed Goose (*Anser brachyrhynchus,* monotypic). It is important to note that this study will focus on the European section of the Bean Goose complex which comprises the subspecies *Anser f. fabalis* and *Anser s. rossicus*. The eastern subspecies (*A. f. johanseni, A. f. middendorfii*, and *A. s. serrirostris*) were not included in the sampling. The taxonomic uncertainty is partly due to the inconsistent phylogenetic relationships within the Bean Goose complex [32–34]. Specifically, different phylogenetic studies have reported different topologies for this complex: analyses of the mitochondrial control region reported a sister species relationship between *A. fabalis* and *A. serrirostris* [33], whereas genome-wide exon-data recovered *A. serrirostris* and *A. brachyrhynchus* as each other’s closest relatives [35]. Widespread occurrence of introgressive hybridization and rapid succession of speciation events in these goose species probably explain these incongruent results [36, 37]. If differentiation islands within the Bean Goose complex have been largely shielded from the misleading effects of selection and introgression, they might have retained the correct species tree.

Above, it has implicitly been assumed that there is a ‘true’ species tree that can be depicted as a bifurcating tree. However, the widespread occurrence of introgression across the Tree of Life challenges this assumption and indicates that a phylogenetic network approach—which takes into account reticulate evolution—might be more appropriate [38–40]. Therefore, we will also quantify the patterns of introgression within the Bean Goose complex to determine whether the evolutionary history of these species can be captured in a bifurcating tree, or if a phylogenetic network might be more suitable. The choice for a phylogenetic tree or a phylogenetic network ultimately depends on aim of the study: reconstructing the order of speciation events calls for a species tree whereas quantifying the impact of introgressive hybridization requires a network approach. In this study, we will apply both perspectives to fully capture the evolutionary history of the Bean Goose complex. In addition, to place our findings in a wider evolutionary context, we also included other closely related goose species in our analyses, namely the Lesser White-fronted Goose (*A. erythropus*), the Greater White-fronted Goose (*A. albifrons*) and the Greylag Goose (*A. anser*). Moreover, we used several species of the genus *Branta* as an outgroup.

## Results

### Sequencing and quality assessment

We collected blood and tissue samples for nine goose taxa (Additional file 1: Table S1): the Taiga Bean Goose (*A. fabalis*, n = 9), the Tundra Bean Goose (*A. serrirostris*, n = 9), the Pink-footed Goose (*A. brachyrhynchus*, n = 15), the Greater White-fronted Goose (*A. albifrons*, n = 10), the Lesser White-fronted Goose (*A. erythropus*, n = 3), the Greylag Goose (*A. anser,* n = 13), the Barnacle Goose (*B. leucopsis*, n = 5), the Canada Goose (*B. canadensis*, n = 2) and the Brent Goose (*B. bernicla,* n = 5). We re-sequenced the genomes of these samples on an Illumina HiSeqX following standard procedures. The resulting reads were mapped to the Swan Goose (*A. cygnoides*) genome (assembled on scaffold level with a genome size of 1.1 Gb, Additional file 1: Table S2) with average mapping percentage of 95.4% (range 83.7–97.9) and an average sequencing depth of 41.4X (range 28.9–77.4).

### Patterns of genetic differentiation

**Fig. 1:**
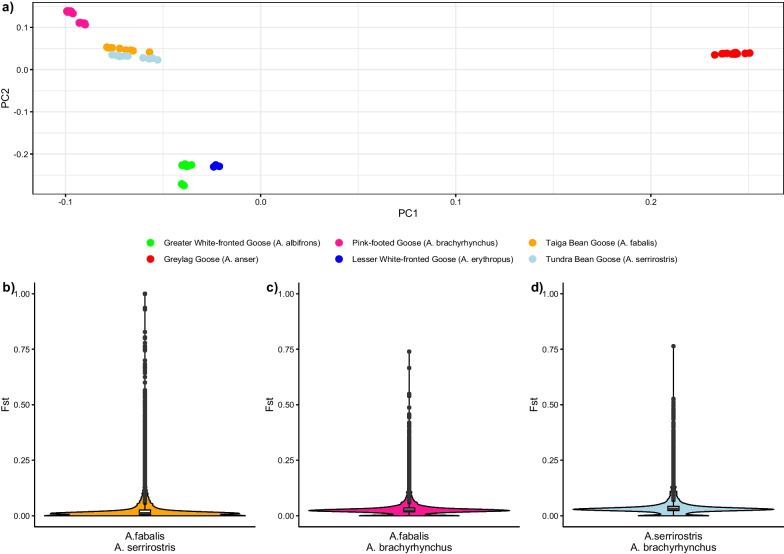
**a** Principal component analysis based on 11,505,116 SNPs discriminates between all goose taxa in this study. Violin plots for different species pairs show that genetic differentiation is concentrated in a few genomic islands: **b** *A. fabalis* and *A. serrirostris*, **c** *A. fabalis* and *A. brachyrhynchus*, and **d** *A. serrirostris* and *A. brachyrhynchus*

We generated a dataset of 11,505,116 SNPs across all sampled goose taxa. A PCA based on this dataset discriminated between all taxa (Fig. 1a): the first principal component mainly separated *A. anser* from all other taxa, but also indicated differences among the remaining five taxa in the genus *Anser*. The second principal component distinguished between the white-fronted geese (*A. albifrons* and *A. erythropus*) and the Bean Goose complex (*A. fabalis*, *A. serrirostris* and *A. brachyrhynchus*). Within the Bean Goose complex, the PCA clearly separated *A. brachyrhynchus* from *A. fabalis* and *A. serrirostris*.

We calculated relative genetic differentiation (*F*_*ST*_) across non-overlapping windows of 200,000 nucleotides (200 kb) using VCFtools version 0.1.15 [41]. These *F*_*ST*_-analyses suggested that genetic differentiation between taxa in the Bean Goose complex can be explained in different ways. Between *A. fabalis* and *A. serrirostris*, most genomic windows showed a low degree of genetic differentiation (genome-wide *F*_*ST*_ = 0.033) and differentiation was concentrated in several windows with high *F*_*ST*_-values (Fig. 1b; 59 *F*_*ST*_-windows > 0.5). *A. brachyrhynchus*, in contrast, was slightly more differentiated from *A. fabalis* (genome-wide *F*_*ST*_ = 0.035) and *A. serrirostris* (genome-wide *F*_*ST*_ = 0.043), but there were fewer high *F*_*ST*_-windows (Fig. 1c, d; 4 and 5 *F*_*ST*_-windows > 0.5, respectively). These discrepancies between the genome-wide differentiation and the distribution of differentiated windows across the genome might explain the difficulty of resolving the phylogenetic relationships within the Bean Goose complex.

### Phylogenetic analyses

**Fig. 2:**
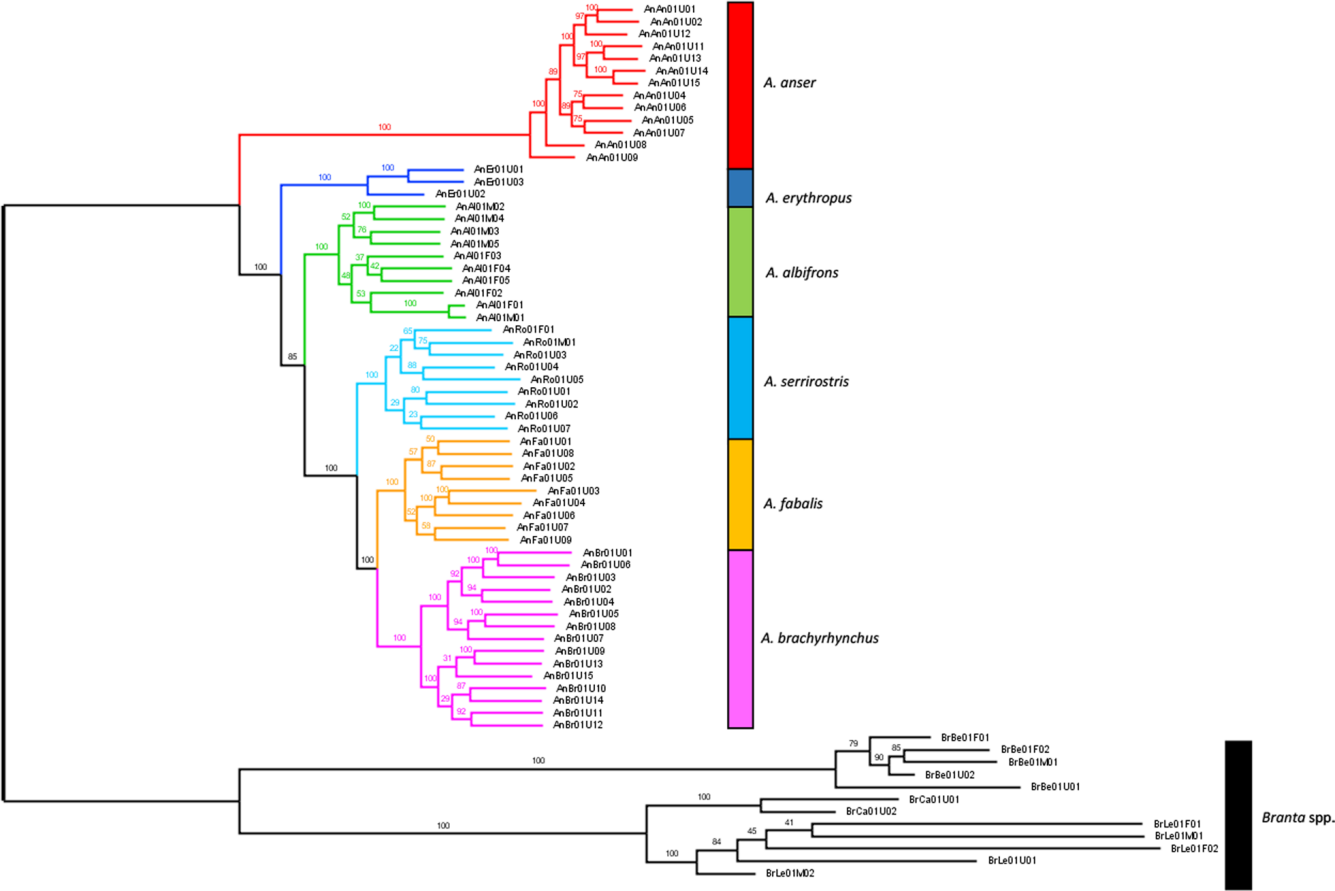
Phylogenetic tree based on a dataset of 2,154,185 high quality SNPs, generated with the TVM + F + R4 substitution model in IQTree 1.5.4. The numbers above the branches indicate statistical support based on 1000 ultrafast bootstraps

To infer phylogenetic relationships among the different goose taxa, we estimated a species tree using a concatenated dataset of 2,154,185 high quality SNPs (see Materials and methods for the selection criteria). This analysis resulted in three monophyletic clades within the Bean Goose complex, with *A. brachyrhynchus* sister to *A. fabalis*, and with *A. serrirostris* sister to the clade containing the latter two species (Fig. 2). This genome-wide phylogeny served as a comparison for more specific phylogenetic analyses using particular selections of genomic windows (i.e. locus trees based on random sampling across the genome versus highly differentiated genomic windows).

**Fig. 3:**
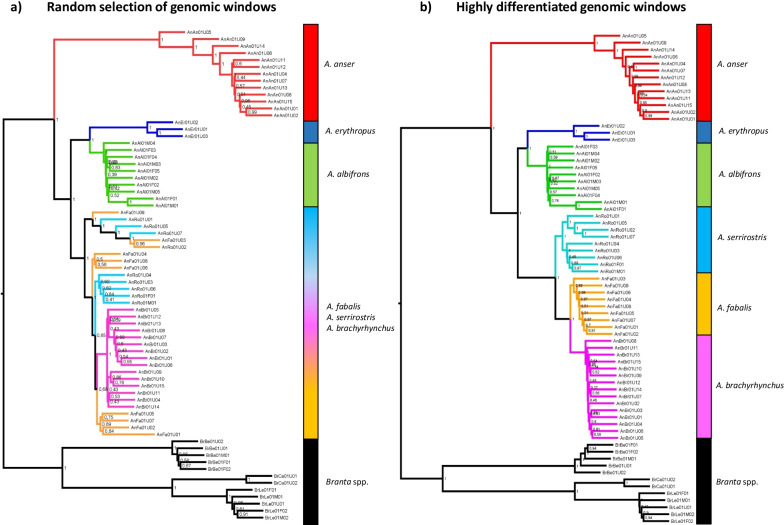
Species tree for **a** a random selection of genomic windows and **b** highly differentiated genomic windows. The different goose taxa are highlighted in different colors. The gradient of colors for *A. fabalis*, *A. serrirostris* and *A. brachyrhynchus* in figure **a** indicates the mixed nature of this clade

**Fig. 4:**
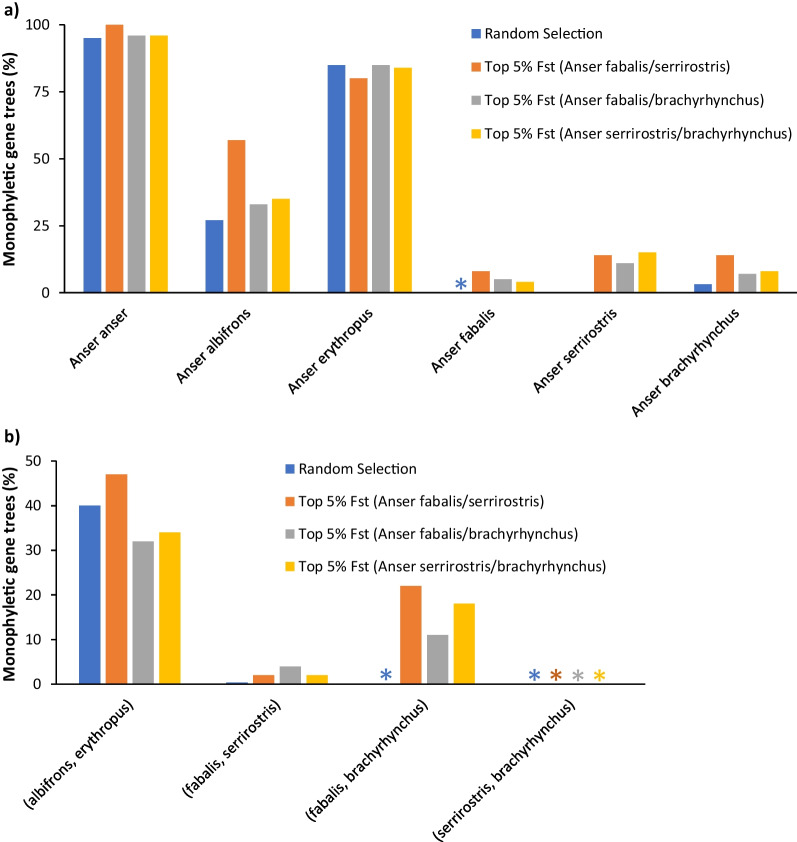
Distribution of locus trees for a random selection of genomic windows and the top 5% differentiated windows for different species combinations. The bar plots show the percentage of monophyletic locus trees for **a** the different goose taxa and **b** combinations of particular goose taxa. Stars (*) indicate cases were no locus trees (0%) supported a particular phylogenetic arrangement

First, we constructed locus trees for genomic windows of 200 kb. Based on the observation of largely undifferentiated genomic landscapes, we hypothesized that the estimation of a species tree from a random selection of genomic windows will not resolve phylogenetic relationships within the Bean Goose complex. This hypothesis was indeed supported by the resulting phylogeny (based on a coalescent analysis with ASTRAL using 500 randomly generated locus trees): a monophyletic *A. brachyrhynchus* clade was nested within a paraphyletic clade containing *A. fabalis* and *A. serrirostris* (Fig. 3a). Close inspection of the underlying locus trees revealed that few of them contained monophyletic clades for the Bean Goose complex (Fig. 4): *A. fabalis* (0% of locus trees), *A. serrirostris* (0.2%) and *A. brachyrhynchus* (3.2%). Interestingly, the relationships between the other goose species were unequivocally resolved. In line with previous phylogenomic work, *A. albifrons* and *A. erythropus* were sister species and *A. anser* was sister to all other *Anser* species in this study. These results were robust to the number of random locus trees used in the species tree analysis (ranging from 50 to 500 genomic windows).

Next, we focused on the highly differentiated windows (defined as the top 5% *F*_*ST*_-windows). For each species pair in the Bean Goose complex, we constructed locus trees for these windows and estimated the species tree from this collection of locus trees with ASTRAL version 5.6.3 [6]. Most analyses converged on the same topology in which the three species form monophyletic clades (Additional file 1: Table S3). *A. brachyrhynchus* is sister to *A. fabalis*, and *A. serrirostris* is sister to the clade containing the latter two species (Fig. 3b). Inspection of the underlying locus trees showed that this topology was the most common (18–22% of locus trees). A sister species relationship between *A. fabalis* and *A. serrirostris* was rarely observed (2–4%), whereas a monophyletic clade with *A. serrirostris* and *A. brachyrhynchus* was never obtained (Fig. 4).

These patterns were even more pronounced when narrowing down to the top 1% *F*_*ST*_-windows (Additional file 1: Table S4). In this case the sister species relationship between *A. fabalis* and *A. brachyrhynchus* was found in 22–41% of the locus trees, while the other topologies were not observed. The relationships between the other goose species remained stable and received maximal support (based on posterior probabilities).

**Fig. 5:**
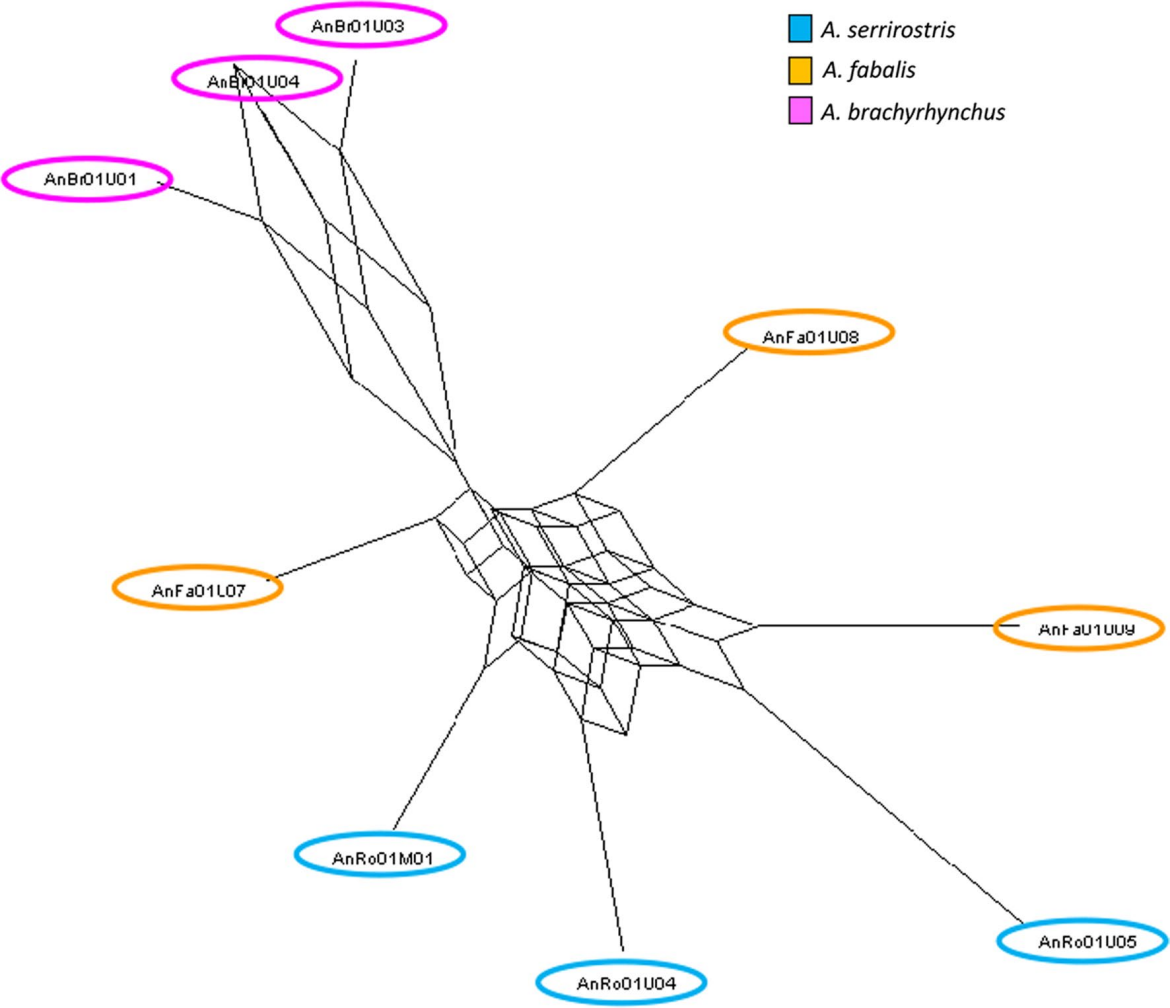
Consensus network of 500 randomly selected locus trees. There is a clear separation between *A. brachyrhynchus* (pink) and the two other species. Moreover, *A. fabalis* (yellow) and *A. serrirostris* (blue) are connected by a complex network and could not be separated into distinct groups

Finally, a phylogenetic network analysis corroborated the patterns described above. The consensus network—based on 500 randomly selected locus trees—showed a clear split between *A. brachyrhynchus* and the other two species. However, individuals of *A. fabalis* and *A. serrirostris* could not be clearly separated and were connected by a complex network (Fig. 5).

### Patterns of introgression

To quantify the influence of introgression on the evolutionary history of the Bean Goose complex, we calculated D-statistics with the program Dtrios [42]. This software orders each trio of taxa so that the ‘BBAA’ pattern is more common than the discordant ‘ABBA’ and ‘BABA’ patterns before computing the D-statistics. This approach resulted in the same topology that we recovered in the phylogenetic analyses of differentiation islands: *A. brachyrhynchus* is most closely related to *A. fabalis*. Calculation of the D-statistics from this phylogenetic arrangement with all *Branta* species as the outgroup suggested introgression between *A. fabalis* and *A. serrirostris* (D = 0.16, Z = 13.2, p < 0.001). The accompanying F4-ratio indicated that 21.9% of the variants showed signatures of introgression between these species (Additional file 1: Table S5).

## Discussion

In this study, we tested the reliability of using genomic regions with different degrees of differentiation to reconstruct the ‘true’ species tree for the Bean Goose complex. First, we applied a random selection of genomic regions, which samples a large undifferentiated section of the genome. This approach did not resolve the Bean Goose complex, but resulted in a monophyletic *A. brachyrhynchus* clade nested within a mixed cluster of *A. fabalis* and *A. serrirostris*. In contrast, phylogenetic analyses of differentiation islands converged upon a topology of three monophyletic clades in which *A. brachyrhynchus* is sister to *A. fabalis*, and *A. serrirostris* is sister to the clade uniting these two species. Interestingly, the phylogenetic relationships between the other species in the genus *Anser* were unaffected by what parts of the genome were used. This observation suggests that differentiation has progressed beyond a certain genomic ‘tipping point’ in those species [43]. In line with previous phylogenomic studies, *A. albifrons* and *A. erythropus* are sister species, and *A. anser* is sister to all *Anser* species in this study [35, 37].

Phylogenetic analyses of differentiation islands are expected to increase the likelihood of monophyletic clades, because the lower effective population size of differentiation islands tends to accelerate the lineage sorting process [27, 44]. As expected, our phylogenetic analyses uncovered more monophyletic clades in differentiation islands compared to a random selection of genomic regions. However, selective sweeps or ancient introgression events might affect the relationships between monophyletic clades and produce a variety of discordant topologies within differentiation islands [28, 29]. Close inspection of the locus trees within differentiation islands did not show a variety of discordant topologies, but instead revealed one dominant phylogenetic arrangement in which *A. brachyrhynchus* is most closely related to *A. fabalis*. It seems unlikely that species-specific selective sweeps or ancient introgression events have impacted all these differentiation islands in the same way. Moreover, phylogenetic analyses based on concatenation of genome-wide SNPs and the calculation of D-statistics converged upon the same species tree. Hence, these findings suggest that differentiation islands might reflect the ‘true’ species tree in the Bean Goose complex. However, it is important to keep in mind that every phylogeny is a hypothesis that remains to be validated by alternative analyses, such as a model-based approach.

However, considering the dominant topology within the differentiation islands as the ‘true’ species tree ignores the strong signatures of introgression between *A. fabalis* and *A. serrirostris*. In a previous study, we found evidence for recent secondary contact (about 60,000 years ago), resulting in high levels of introgression from *A. serrirostris* into *A. fabalis* [45]. These introgression events have probably impacted the phylogenetic relationships at certain genomic regions. In combination with the large effective population sizes of these goose taxa (see [37]), which implies high levels of incomplete lineage sorting, introgression patterns partly explain the failure to resolve the Bean Goose complex from a random selection of genomic regions. A possible scenario entails that after the divergence between *A. fabalis* and *A. serrirostris*, a population of *A. fabalis* became geographically isolated and colonized several islands (e.g., Svalbard, Greenland or Iceland), ultimately giving rise to *A. brachyrhynchus* [46]. Later on, extensive hybridization between *A. fabalis* and *A. serrirostris* erased the phylogenetic branching pattern between these taxa, resulting in a mixed clade of *A. fabalis* and *A. serrirostris* containing a monophyletic *A. brachyrhynchus* [45]. Differentiation islands were largely unaffected by homogenizing introgression—perhaps because they contained loci involved in reproductive isolation—and maintained the phylogenetic patterns that reflect the species tree.

**Fig. 6:**
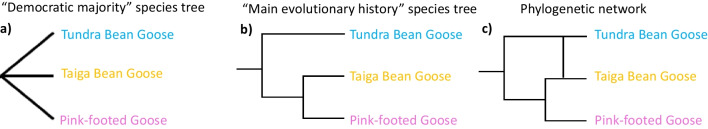
An overview of different ways to represent the evolutionary history of the Bean Goose complex: **a** based on the democratic majority of the genome, **b** reflecting the main evolutionary history according to differentiation islands, or **c** as a phylogenetic network to account for introgression

Here, our study touches upon a philosophical question: what is the species tree? Some authors have proposed that the species tree represents the ‘democratic majority’ of the genome [3], while others argued that the species tree depicts the main diversification history regardless of the genomic proportion supporting it [38]. In our study, the ‘democratic majority’ species tree would depict an unresolved Bean Goose complex reflected by the genome-wide phylogeny based on a random selection of genomic regions (Fig. 6a). The species tree generated from a small set of highly differentiated windows likely represents the dominant evolutionary history of the Bean Goose complex (Fig. 6b). However, depicting the phylogenetic relationships between these taxa as a simple bifurcating tree ignores recent introgression dynamics between *A. fabalis* and *A. serrirostris*. Hence, the evolutionary history of the Bean Goose complex might be better represented as a phylogenetic network that illustrates the reticulate nature of their evolution (Fig. 6c, see also [37, 47]).

Finally, these findings could also inform the taxonomy of the Bean Goose complex, specifically the species status of *A. fabalis* and *A. serrirostris*. Some authors have argued that they should be classified as distinct species [32], while others recommended a classification as subspecies [45]. The phylogenetic position of *A. brachyrhynchus*—which seems to be most closely related to *A. fabalis*—indicates that *A. fabalis* and *A. serrirostris* should be treated as separate taxa to avoid paraphyletic groupings. If one wants to delineate monophyletic clades, all three taxa should thus be classified as either species or subspecies (although some taxonomists do not object to lumping non-sister clades, see [48]). A thorough taxonomic analysis, including eastern taxa of the Bean Goose complex (e.g., Middendorf's Bean Goose), is warranted to achieve a consensus regarding the (sub)species status of the different taxa within this species complex.

## Conclusion

Using whole-genome re-sequencing data, we showed that genetic differentiation between *A. fabalis*, *A. serrirostris* and *A. brachyrhynchus* is concentrated in a few genomic regions whereas the rest of the genome is largely undifferentiated. The uncovered genomic landscape of differentiation informed our subsequent phylogenomic analyses. First, we showed that a random selection of locus trees across the genome—which mainly samples undifferentiated loci—results in an unresolved species complex. Next, we focused on highly differentiated regions to resolve the relationships within the Bean Goose complex, showing that *A. fabalis* is sister to *A. brachyrhynchus*. This topology was not supported across the genome, probably because recent introgression between *A. fabalis* and *A. serrirostris* has erased the phylogenetic branching pattern at certain genomic loci. Differentiation islands appear to have been largely unaffected by the homogenizing introgression and have maintained the phylogenetic branching patterns that reflect the species tree. Because depicting the phylogenetic relationships within the Bean Goose complex as a simple bifurcating tree ignores the recent introgression dynamics between *A. fabalis* and *A. serrirostris*, we advocate that the evolutionary history of this species complex is best represented as a phylogenetic network.

## Materials and methods

### Sequencing and quality assessment

We collected blood and tissue samples for nine goose taxa (Additional file 1: Table S1): the Taiga Bean Goose (*A. fabalis*, n = 9), the Tundra Bean Goose (*A. serrirostris*, n = 9), the Pink-footed Goose (*A. brachyrhynchus*, n = 15), the Greater White-fronted Goose (*A. albifrons*, n = 10), the Lesser White-fronted Goose (*A. erythropus*, n = 3), the Greylag Goose (*A. anser,* n = 13), the Barnacle Goose (*B. leucopsis*, n = 5), the Canada Goose (*B. canadensis*, n = 2) and the Brent Goose (*B. bernicla,* n = 5). Genomic DNA was isolated from these samples using a Qiagen Gentra kit (Qiagen Inc.). Quality and quantity of the DNA was measured using a Qubit (Invitrogen, Life Technologies).

Sequencing libraries were prepared from 100 ng of DNA using the TruSeq Nano DNA sample preparation kit (cat# FC-121-4001/4002, Illumina Inc.) targeting an insert size of 350 bp. Paired-end sequencing (150 bp) was performed on an Illumina HiSeqX following standard procedures. Sequencing reads were mapped to the Swan Goose (*A. cygnoides*) genome version 1.0 [49] using Burrows–Wheeler Aligner (BWA) version 0.7.17 [50]. The resulting BAM-files were sorted with samtools version 1.6 [51] and duplicates were marked with Picard version 2.10.3  (http://broadinstitute.github.io/picard/). Next, local realignment was performed using GATK version 3.7 [52]. For each individual, a first round of variant calling was performed with GATK HaplotypeCaller. The resulting list of variants was filtered based on mapping quality (MQRankSum < 0.22) and read depth (DP > 10). The variants passing these filters were then used as a reference set for base quality score recalibration (BQSR) following a bootstrapping approach in GATK. Next, we applied a hard filter in line with the GATK best practices pipeline [53], applying the following filtering criteria: QD < 2.0 || FS > 60.0 || MQ < 40.0 || MQRankSum < − 12.5 || ReadPosRankSum < − 8.0.

### Phylogenetic analyses

Using VCFtools version 0.1.15 [41], we removed loci for which the p-value was smaller than 0.01 in a test for excess of heterozygotes relative to Hardy–Weinberg genotype proportions. Moreover, we retained only loci with a minor allele frequency ≥ 0.05. Finally, the SNPs were filtered based on linkage disequilibrium along windows of 50 markers with a R^2^-threshold of 0.5. The resulting dataset of 11,505,116 SNPs provided the input for principal component analysis (PCA) using the pca-function in Plink version 1.07 [54]. Visualizing the samples in a PCA is quick and reliable way to identify any outliers or mistakes before running more computer-intensive analyses. Next, we constructed the genomic landscape of differentiation for all pairwise species combinations by calculating relative genetic differentiation (*F*_*ST*_) across non-overlapping windows of 200,000 nucleotides (200 kb) using VCFtools version 0.1.15 [41]. We opted for a window size of 200 kb because this allowed us to directly compare the resulting patterns with previous work [45]. Moreover, larger windows sizes (> 100 kb) are often more reliable in estimating phylogenetic relationships between recently diverged populations [55, 56].

We converted the VCF-files into Fasta-format using scripts available at https://github.com/edgardomortiz/vcf2phylip. We estimated a phylogenetic tree based on a concatenated dataset of 2,154,185 high quality SNPs—based on the procedure described in the previous paragraph—that were present in 66 out of 71 individuals. The model selection (without ascertainment bias correction) in IQTree 1.5.4 [58] recommended the TVM + F + R4 substitution model. Hence, we ran IQTree 1.5.4 [57] with this model and included 1000 ultrafast bootstraps [59].

Next, we constructed locus trees for differentiated 200 kb windows (top 5% and top 1% *F*_*ST*_-outliers) from different species combinations. The resulting number of differentiated windows ranged between 228 and 328 for the top 5% *F*_*ST*_-outliers, and between 46 and 50 for the top 1% *F*_*ST*_-outliers. Locus trees were constructed using IQTree 1.5.4 [57] with model selection [58] and 1000 ultrafast bootstraps [59]. For each set of locus trees, we estimated a species tree using ASTRAL version 5.6.3 [6]. ASTRAL is consistent with the multispecies coalescent and takes into account incomplete lineage sorting when estimating the species tree from a collection of locus trees. Moreover, the multispecies coalescent is quite robust to variation in intralocus recombination with little impact on the estimation of species trees [60]. Hence, we could use relatively long genomic segments (200 kb) that contain sufficient substitutions to resolve phylogenetic relationships in the locus trees. The reliability of the resulting species tree was assessed by Bayesian posterior probabilities that are computed with a quartet-based method [61]. The resulting species trees were edited with FigTree version 4.1.3 (http://tree.bio.ed.ac.uk/software/figtree/). As a control, we also inferred phylogenetic relationships from several random selections of genomic windows (from 50 to 500 windows), following the procedure outlined above. Finally, we quantified the distribution of tree topologies and calculated the proportion of monophyletic clades for all locus trees using the is.monophyletic-function in the R-package *ape* version 5 [62].

In addition to the phylogenetic tree analyses, we estimated a phylogenetic network based on a random selection of 500 locus trees. We used the “Consensus Network” method in SplitsTree v.4.18.3 [63] with a minimum proportion of trees supporting the splits of 0.1. To improve the visibility of the resulting network, three individuals per taxon were randomly selected. Several runs revealed that the choice of individuals did not affect the overall topology of the network.

### Patterns of introgression

We calculated introgression rates between members of the Bean Goose complex using the Dtrios program of the Dsuite software package [42], which makes no prior assumptions about the phylogenetic relationships between the included taxa other than the outgroup. Dtrios orders each trio of taxa under the assumption that the correct tree is the one where the ‘BBAA’ pattern is more common than the discordant ‘ABBA’ and ‘BABA’ patterns, which can be the outcome of incomplete lineage sorting or introgression. To assess whether a D-statistic is significantly different from zero, Dtrios uses a standard block-jackknife procedure which provides a Z-score and an associated p-value [64]. These analyses allowed us to estimate how strongly introgressive hybridization has impacted the evolutionary history of the Bean Goose complex.

## Supplementary Information

**Additional file 1. Table S1.** Overview of sampling locations. **Table S2.** Mapping and coverage statistics for all samples in this study. **Table S3.** Outcomes of phylogenetic analyses for different selections of highly differentiated genomic windows. **Table S4.** Percentage of monophyletic gene trees for the different goose taxa and particular combinations of taxa. **Table S5.** Detailed information on the D-statistics analysis of Dtrios.

## Data Availability

The genome re-sequencing data are freely available in EMBL‐EBI European Nucleotide Archive (http://www.ebi.ac.uk/ena) under accession number PRJEB35788. The scripts and workflow for the analyses can be found on the following Github-page: https://github.com/JenteOttie/Goose_Genomics/.
