# Supplementary material for: Highly differentiated loci resolve phylogenetic relationships in the Bean Goose complex
Source: BMC Ecol Evol. 2023 Jan 19;23:2. doi: 10.1186/s12862-023-02103-3 (PMC9854053; doi:10.1186/s12862-023-02103-3)
Supplement: Supplementary file 1 — Additional file 1. Table S1. Overview of sampling locations. Table S2. Mapping and coverage statistics for all samples in this study. Table S3. Outcomes of phylogenetic analyses for different selections of highly differentiated genomic windows. Table S4. Percentage of monophyletic gene trees for the different goose taxa and particular combinations of taxa. Table S5. Detailed information on the D-statistics analysis of Dtrios. [file 12862_2023_2103_MOESM1_ESM.docx]

**Supplementary Material: Highly differentiated loci resolve phylogenetic relationships in the Bean Goose complex**

Jente Ottenburghs^1^, Johanna Honka^2^, Marja E. Heikkinen^2^, Jesper Madsen^3^, Gerhard J.D.M. Müskens^4^ & Hans Ellegren^1^

^1^ Department of Evolutionary Biology, University of Uppsala, Uppsala, Sweden.

^2^ Department of Ecology and Genetics, University of Oulu, PO Box 3000, FI-90014, Oulu, Finland.

^3^ Department of Bioscience – Kalø, Aarhus University, Grenåvej 14, 8410, Rønde, Denmark.

^4^ Team Animal Ecology, Wageningen Environmental Research, Wageningen University & Research, Droevendaalsesteeg 3-3A, 6708 PB, Wageningen, The Netherlands.

**Contents**

Table S1. Overview of sampling locations.

Table S2. Mapping and coverage statistics for all samples in this study

Table S3. Outcomes of phylogenetic analyses for different selections of highly differentiated genomic windows.

Table S4. Percentage of monophyletic gene trees for the different goose taxa and particular combinations of taxa.

Table S5. Detailed information on the D-statistics analysis of Dtrios.

**Table S1**. Information about sampling locations for all goose specimens in this study.

| **SampleName** | **Code** | **Species** | **Collection** | **Location** | **Country** |
| --- | --- | --- | --- | --- | --- |
| AnAl01F01 | 255-443 | *A. albifrons* | G. Müskens | Maren-Kessel | The Netherlands |
| AnAl01F02 | 255-449 | *A. albifrons* | G. Müskens | Lith | The Netherlands |
| AnAl01F03 | 255-491 | *A. albifrons* | G. Müskens | Kollumerland | The Netherlands |
| AnAl01F04 | 283-290 | *A. albifrons* | G. Müskens | Maren-Kessel | The Netherlands |
| AnAl01F05 | 283-253 | *A. albifrons* | G. Müskens | Maren-Kessel | The Netherlands |
| AnAl01M01 | 255-447 | *A. albifrons* | G. Müskens | Maren-Kessel | The Netherlands |
| AnAl01M02 | 255-478 | *A. albifrons* | G. Müskens | Lith | The Netherlands |
| AnAl01M03 | 255-481 | *A. albifrons* | G. Müskens | Kollumerland | The Netherlands |
| AnAl01M04 | 283-281 | *A. albifrons* | G. Müskens | Maren-Kessel | The Netherlands |
| AnAl01M05 | 283-241 | *A. albifrons* | G. Müskens | Maren-Kessel | The Netherlands |
| AnAn01U01 | NRM 976348 | *A. anser* | Museum Stockholm | Uppland | Sweden |
| AnAn01U02 | NRM 20006278 | *A. anser* | Museum Stockholm | Öland | Sweden |
| AnAn01U04 | IR1 | *A. anser* | M. Heikkinen | Gilan | Iran |
| AnAn01U05 | IR2 | *A. anser* | M. Heikkinen | Gilan | Iran |
| AnAn01U06 | IR3 | *A. anser* | M. Heikkinen | Fereydunkenar | Iran |
| AnAn01U07 | IR4 | *A. anser* | M. Heikkinen | Fereydunkenar | Iran |
| AnAn01U08 | KZ1 | *A. anser* | M. Heikkinen | Kulykol | Kazakhstan |
| AnAn01U09 | GR1 | *A. anser* | M. Heikkinen | Miliónas | Greece |
| AnAn01U11 | FI9 | *A. anser* | M. Heikkinen | Ylöjärvi | Finland |
| AnAn01U12 | FI13 | *A. anser* | M. Heikkinen | Hailuoto | Finland |
| AnAn01U13 | FI18 | *A. anser* | M. Heikkinen | Kimito | Finland |
| AnAn01U14 | FI44 | *A. anser* | M. Heikkinen | Lumijoki | Finland |
| AnAn01U15 | FI49 | *A. anser* | M. Heikkinen | Mustasaari | Finland |
| AnBr01U01 | 206 | *A. brachyrhynchus* | J. Madsen | Vest Stadil Fjord | Denmark |
| AnBr01U02 | 12 | *A. brachyrhynchus* | J. Madsen | Vest Stadil Fjord | Denmark |
| AnBr01U03 | 215 | *A. brachyrhynchus* | J. Madsen | Vest Stadil Fjord | Denmark |
| AnBr01U04 | 216 | *A. brachyrhynchus* | J. Madsen | Vest Stadil Fjord | Denmark |
| AnBr01U05 | 126 | *A. brachyrhynchus* | J. Madsen | Vest Stadil Fjord | Denmark |
| AnBr01U06 | 177 | *A. brachyrhynchus* | J. Madsen | Vest Stadil Fjord | Denmark |
| AnBr01U07 | 147 | *A. brachyrhynchus* | J. Madsen | Vest Stadil Fjord | Denmark |
| AnBr01U08 | 172 | *A. brachyrhynchus* | J. Madsen | Vest Stadil Fjord | Denmark |
| AnBr01U09 | 330 | *A. brachyrhynchus* | J. Madsen | Vest Stadil Fjord | Denmark |
| AnBr01U10 | 340 | *A. brachyrhynchus* | J. Madsen | Vest Stadil Fjord | Denmark |
| AnBr01U11 | 320 | *A. brachyrhynchus* | J. Madsen | Vest Stadil Fjord | Denmark |
| AnBr01U12 | 303 | *A. brachyrhynchus* | J. Madsen | Vest Stadil Fjord | Denmark |
| AnBr01U13 | 341 | *A. brachyrhynchus* | J. Madsen | Vest Stadil Fjord | Denmark |
| AnBr01U14 | 306 | *A. brachyrhynchus* | J. Madsen | Vest Stadil Fjord | Denmark |
| AnBr01U15 | 322 | *A. brachyrhynchus* | J. Madsen | Vest Stadil Fjord | Denmark |
| AnEr01U01 | NRM 20036732 | *A. erythropus* | Museum Stockholm | NA | Sweden |
| AnEr01U02 | NRM 996391 | *A. erythropus* | Museum Stockholm | Captive Bird | Sweden |
| AnEr01U03 | NRM 20106538 | *A. erythropus* | Museum Stockholm | Hälsingland | Sweden |
| AnFa01U01 | NRM 20006276 | *A. fabalis* | Museum Stockholm | Kristianstad, Trolle-Ljungby | Sweden |
| AnFa01U02 | NRM 20006268 | *A. fabalis* | Museum Stockholm | NA | Sweden |
| AnFa01U03 | 20A2012 | *A. fabalis* | J. Honka | Lokka Tekoallas | Finland |
| AnFa01U04 | 25C2012 | *A. fabalis* | J. Honka | Liminka | Finland |
| AnFa01U05 | 112012 | *A. fabalis* | J. Honka | Selänpää | Finland |
| AnFa01U06 | 232012 | *A. fabalis* | J. Honka | Kankaanpää | Finland |
| AnFa01U07 | 242012 | *A. fabalis* | J. Honka | Pudasjärvi | Finland |
| AnFa01U08 | 33B2012 | *A. fabalis* | J. Honka | Vaala | Finland |
| AnFa01U09 | 32A2012 | *A. fabalis* | J. Honka | Toholampi | Finland |
| AnRo01F01 | 255-349 | *A. serrirostris* | G. Müskens | Maren-Kessel | The Netherlands |
| AnRo01M01 | 255-346 | *A. serrirostris* | G. Müskens | Maren-Kessel | The Netherlands |
| AnRo01U01 | 362012 | *A. serrirostris* | J. Honka | Kittilä | Finland |
| AnRo01U02 | 102012 | *A. serrirostris* | J. Honka | Rääkkylä | Finland |
| AnRo01U03 | 192012 | *A. serrirostris* | J. Honka | Hamina | Finland |
| AnRo01U04 | 14B2012 | *A. serrirostris* | J. Honka | Virolahti | Finland |
| AnRo01U05 | 382012 | *A. serrirostris* | J. Honka | Tyrnävä | Finland |
| AnRo01U06 | 22B2012 | *A. serrirostris* | J. Honka | Joutseno | Finland |
| AnRo01U07 | 412012 | *A. serrirostris* | J. Honka | Salo | Finland |
| BrBe01F01 | 283-357 | *B. bernicla* | G. Müskens | Terschelling-Strieperpolder | The Netherlands |
| BrBe01F02 | 283-380 | *B. bernicla* | G. Müskens | Terschelling-Strieperpolder | The Netherlands |
| BrBe01M01 | 283-397 | *B. bernicla* | G. Müskens | Terschelling-Strieperpolder | The Netherlands |
| BrBe01U01 | NRM 986368 | *B. bernicla* | Museum Stockholm | Öland | Sweden |
| BrBe01U02 | NRM 20026465 | *B. bernicla* | Museum Stockholm | Öland | Sweden |
| BrCa01U01 | NRM 996396 | *B. canadensis* | Museum Stockholm | Uppland | Sweden |
| BrCa01U02 | NRM 20036930 | *B. canadensis* | Museum Stockholm | Uppland | Sweden |
| BrLe01F01 | 255-494 | *B. leucopsis* | G. Müskens | Kollumerland | The Netherlands |
| BrLe01F02 | 267-266 | *B. leucopsis* | G. Müskens | Maren-Kessel | The Netherlands |
| BrLe01M01 | 255-520 | *B. leucopsis* | G. Müskens | Kollumerland | The Netherlands |
| BrLe01M02 | 267-283 | *B. leucopsis* | G. Müskens | Maren-Kessel | The Netherlands |
| BrLe01U01 | NRM 946130 | *B. leucopsis* | Museum Stockholm | Gotland | Sweden |

**Table S2.** Mapping and coverage statistics for all samples in this study

| **Sample** | **Number of reads** | **Mapped reads** | **Mean Coverage** |
| --- | --- | --- | --- |
| AnAl01F01 | 346 817 434 | 331 400 665 (95.6%) | 41.87X |
| AnAl01F02 | 311 587 909 | 292 839 683 (94.0%) | 36.15X |
| AnAl01F03 | 255 867 691 | 244 805 388 (95.7%) | 30.99X |
| AnAl01F04 | 377 367 192 | 349 331 344 (92.6%) | 42.37X |
| AnAl01F05 | 325 280 182 | 310 404 047 (95.4%) | 39.10X |
| AnAl01M01 | 328 585 003 | 314 142 628 (95.6%) | 39.70X |
| AnAl01M02 | 297 279 901 | 282 627 104 (95.1%) | 35.52X |
| AnAl01M03 | 380 954 523 | 365 626 900 (96.0%) | 46.54X |
| AnAl01M04 | 333 925 784 | 318 977 625 (95.5%) | 39.98X |
| AnAl01M05 | 324 661 600 | 310 580 775 (95.7%) | 39.46X |
| AnAn01U01 | 302 975 457 | 294 203 438 (97.1%) | 37.51X |
| AnAn01U02 | 306 074 503 | 297 008 453 (97.0%) | 37.77X |
| AnAn01U04 | 402 656 051 | 353 797 370 (87.9%) | 39.75X |
| AnAn01U05 | 286 840 154 | 251 209 798 (87.6%) | 28.89X |
| AnAn01U06 | 369 007 833 | 317 736 456 (86.1%) | 36.02X |
| AnAn01U07 | 312 711 620 | 276 133 408 (88.3%) | 31.77X |
| AnAn01U08 | 307 292 856 | 285 162 277 (92.8%) | 32.09X |
| AnAn01U09 | 354 346 844 | 314 707 968 (88.8%) | 34.94X |
| AnAn01U11 | 391 836 426 | 355 097 980 (90.6%) | 42.48X |
| AnAn01U12 | 270 016 886 | 249 853 535 (92.5%) | 29.14X |
| AnAn01U13 | 350 105 012 | 331 359 033 (94.7%) | 39.35X |
| AnAn01U14 | 410 059 083 | 375 087 163 (91.5%) | 41.94X |
| AnAn01U15 | 369 822 941 | 349 451 889 (94.5%) | 43.94X |
| AnBr01U01 | 623 561 810 | 606 998 625 (97.3%) | 75.90X |
| AnBr01U02 | 352 230 699 | 344 644 282 (97.8%) | 44.13X |
| AnBr01U03 | 344 081 243 | 334 827 047 (97.3%) | 41.72X |
| AnBr01U04 | 593 489 473 | 578 271 578 (97.4%) | 72.01X |
| AnBr01U05 | 798 862 768 | 778 779 644 (97.5%) | 77.43X |
| AnBr01U06 | 637 174 223 | 621 199 985 (97.5%) | 75.95X |
| AnBr01U07 | 614 522 367 | 599 822 929 (97.6%) | 50.87X |
| AnBr01U08 | 451 552 018 | 433 932 479 (96.1%) | 32.25X |
| AnBr01U09 | 256 117 803 | 250 030 585 (97.6%) | 39.42X |
| AnBr01U10 | 327 091 149 | 318 527 749 (97.4%) | 39.96X |
| AnBr01U11 | 328 644 098 | 319 629 518 (97.3%) | 49.01X |
| AnBr01U12 | 402 958 797 | 384 580 817 (95.4%) | 29.68X |
| AnBr01U13 | 239 419 817 | 233 024 685 (97.3%) | 38.73X |
| AnBr01U14 | 315 180 459 | 306 434 080 (97.2%) | 39.32X |
| AnBr01U15 | 324 952 728 | 314 832 246 (96.9%) | 38.00X |
| AnEr01U01 | 310 722 211 | 300 164 969 (96.6%) | 40.71X |
| AnEr01U02 | 333 460 228 | 321 349 024 (96.4%) | 37.16X |
| AnEr01U03 | 299 889 432 | 290 614 376 (96.9%) | 75.90X |
| AnFa01U01 | 312 362 054 | 304 812 645 (97.58%) | 38.30X |
| AnFa01U02 | 325 099 485 | 314 649 926 (96.79%) | 39.58X |
| AnFa01U03 | 387 180 588 | 359 734 515 (92.91%) | 40.05X |
| AnFa01U04 | 313 424 653 | 262 454 848 (83.74%) | 30.73X |
| AnFa01U05 | 322 101 618 | 305 479 772 (94.84%) | 37.62X |
| AnFa01U06 | 392 536 125 | 365 170 541 (93.03%) | 42.99X |
| AnFa01U07 | 340 473 167 | 325 662 754 (95.65%) | 38.84X |
| AnFa01U08 | 326 353 112 | 302 622 885 (92.73%) | 36.00X |
| AnFa01U09 | 371 017 606 | 334 170 460 (90.07%) | 38.41X |
| AnRo01F01 | 319 819 026 | 308 937 936 (96.6%) | 38.90X |
| AnRo01M01 | 363 607 500 | 352 523 736 (96.95%) | 43.93X |
| AnRo01U01 | 358 627 964 | 318 316 595 (88.76%) | 35.36X |
| AnRo01U02 | 344 639 151 | 310 242 537 (90.02%) | 34.87X |
| AnRo01U03 | 338 470 668 | 317 514 405 (93.81%) | 36.84X |
| AnRo01U04 | 369 079 405 | 337 993 551 (91.58%) | 39.45X |
| AnRo01U05 | 346 764 567 | 312 937 204 (90.24%) | 32.71X |
| AnRo01U06 | 304 353 937 | 277 986 328 (91.34%) | 33.57X |
| AnRo01U07 | 329 727 996 | 299 965 175 (90.97%) | 34.74X |
| BrBe01F01 | 411 036 995 | 384 307 149 (93.5%) | 45.03X |
| BrBe01F02 | 406 334 698 | 387 361 413 (95.3%) | 46.80X |
| BrBe01M01 | 310 143 965 | 298 654 710 (96.3%) | 37.03X |
| BrBe01U01 | 306 617 855 | 297 487 482 (97.0%) | 37.33X |
| BrBe01U02 | 347 973 362 | 338 785 970 (97.4%) | 43.22X |
| BrCa01U01 | 337 933 463 | 327 225 691 (96.8%) | 40.81X |
| BrCa01U02 | 336 667 954 | 327 162 939 (97.2%) | 41.06X |
| BrLe01F01 | 301 759 057 | 288 157 799 (95.5%) | 36.02X |
| BrLe01F02 | 441 038 804 | 422 635 440 (95.8%) | 52.84X |
| BrLe01M01 | 297 235 489 | 282 151 907 (94.9%) | 34.84X |
| BrLe01M02 | 597 983 811 | 576 911 348 (96.5%) | 72.14X |
| BrLe01U01 | 410 054 512 | 395 502 185 (96.5%) | 49.29X |

**Table S3.** Outcomes of phylogenetic analyses for different selections of highly differentiated genomic windows.

| **Summary Statistic** | **Species**  **Combination** | **Threshold** | **Number of Gene Trees** | **Topology** |
| --- | --- | --- | --- | --- |
| Fst | *A. brachyrhynchus – A. fabalis* | Top 5% | 328 | ((*brachyrhynchus, fabalis*), *serrirostris*) |
|  |  | Top 1% | 50 | ((*brachyrhynchus, fabalis*), *serrirostris*) |
| Fst | *A. brachyrhynchus – A. serrirostris* | Top 5% | 228 | ((*brachyrhynchus, fabalis*), *serrirostris*) |
|  |  | Top 1% | 46 | ((*brachyrhynchus + fabalis*), *serrirostris*)^1^ |
| Fst | *A. serrirostris – A. fabalis* | Top 5% | 250 | ((*brachyrhynchus, fabalis*), *serrirostris*) |
|  |  | Top 1% | 49 | ((*brachyrhynchus, fabalis*), *serrirostris*) |

^1^ A monophyletic *A. brachyrhynchus* clade embedded within *A. fabalis*.

**Table S4.** Percentage of monophyletic gene trees for the different goose taxa and particular combinations of taxa.

|  | **Random Trees** | **Top 1% differentiation islands** | | | **Top 5% differentiation islands** | | |
| --- | --- | --- | --- | --- | --- | --- | --- |
| **SPECIES** |  | *fabalis*  *serrirostris* | *brachyrhynchus*  *fabalis* | *brachyrhynchus*  *serrirostris* | *fabalis*  *serrirostris* | *brachyrhynchus*  *fabalis* | *brachyrhynchus*  *serrirostris* |
| *Anser anser* | 95 | 100 | 100 | 100 | 100 | 96 | 96 |
| *Anser albifrons* | 27 | 45 | 56 | 48 | 57 | 33 | 35 |
| *Anser erythropus* | 85 | 84 | 90 | 78 | 80 | 85 | 84 |
| *Anser fabalis* | 0 | 10 | 16 | 0 | 8 | 5 | 4 |
| *Anser serrirostris* | 0,2 | 18 | 12 | 24 | 14 | 11 | 15 |
| *Anser brachyrhynchus* | 3,2 | 10 | 6 | 13 | 14 | 7 | 8 |
|  |  |  |  |  |  |  |  |
| **CLADES** |  |  |  |  |  |  |  |
| *(albifrons, erythropus)* | 40 | 59 | 38 | 41 | 47 | 32 | 34 |
| *(fabalis, serrirostris)* | 0,4 | 0 | 0 | 0 | 2 | 4 | 2 |
| *(fabalis, brachyrhynchus)* | 0 | 41 | 22 | 33 | 22 | 11 | 18 |
| *(serrirostris, brachyrhynchus)* | 0 | 0 | 0 | 0 | 0 | 0 | 0 |

**Table S5.** Detailed information on the D-statistics analysis of Dtrios.

| **P1** | **P2** | **P3** | **O** | **ABBA** | **BABA** | **D** | **Z** | **F4-ratio** | **Interpretation** |
| --- | --- | --- | --- | --- | --- | --- | --- | --- | --- |
| *A. brachyrhynchus* | *A. fabalis* | *A. serrirostris* | *Branta* species | 840 145 | 814 211 | 0.016 | 13.2 | 0.21 | *A. fabalis ↔ A. serrirostris* |
